# Pharmacological blockade of KV1.3 channel as a promising treatment in autoimmune diseases

**DOI:** 10.1016/j.jtauto.2022.100146

**Published:** 2022-01-24

**Authors:** Carlos A. Cañas, Santiago Castaño-Valencia, Fernando Castro-Herrera

**Affiliations:** a Universidad Icesi, CIRAT, Centro de Investigación en Reumatología, Autoinmunidad y Medicina Traslacional, Cali, Colombia; b Unit of Rheumatology, Fundación Valle del Lili, Cali, Colombia; c Department of Physiological Sciences, Department of Health Sciences, Universidad del Valle, Cali, Colombia

**Keywords:** Voltage-gated ion channel, T lymphocyte, Autoimmune channelopathies, Scorpion toxins, Anemone toxin, Immunosuppressor

## Abstract

There are more than 100 autoimmune diseases (AD), which have a high prevalence that ranges between 5% and 8% of the general population. Type I diabetes mellitus, multiple sclerosis, systemic lupus erythematosus and rheumatoid arthritis remain the health problem of highest concern among people worldwide due to its high morbidity and mortality. The development of new treatment strategies has become a research hotspot. In recent years, the study of the ion channels presents in the cells of the immune system, regarding their functional role, the consequences of mutations in their genes and the different ways of blocking them are the subject of intense research. Pharmacological blockade of KV1.3 channel inhibits Ca2+ signaling, T cell proliferation, and pro-inflammatory interleukins production in human CD4^+^ effector memory T cells. These cells mediated most of the AD and their inhibition is a promising therapeutic target. In this review, we will highlight the biological function of KV1.3 channel in T cells, consequence of the pharmacological inhibition (through anemone and scorpion toxins, synthetic peptides, nanoparticles, or monoclonal antibodies) as well as the possible therapeutical application in AD.

## 1 Introduction

The immune system can recognize its own antigens and not establish a response against them, this process is known as antigenic tolerance. When antigenic tolerance is lost and an immune response against it is created, autoimmunity and autoimmune diseases (ADs) are constituted [1]. There are more than 100 ADs, which have a high prevalence that ranges between 5% and 8% of the general population, with a higher presence in women [2]. ADs are a significant clinical problem, due to their complexity and chronicity. Its etiology is multifactorial, among which genetic, epigenetic, environmental, and infectious aspects stand out [3]. Once the disease is established, various changes in the cells of the immune system occur; being of special importance those that are presented in T lymphocytes (T cells) and B lymphocytes (B cell) [4]. In recent years, the study of the ion channels presents in these cells and their role in immune function, are subject of intense research [5], particularly KV1.3 and KCa1.3 channels which participate in regulation of Ca2+ signaling to induce T-cell proliferation and activation, and cytokine production [6]. Effector memory T cells (TEM cells), which are very relevant in the pathogenesis of ADs, may be inhibited through the blocking of KV1.3 channels on the cellular membrane [7]. Blocking of KV1.3 channels from in vitro studies with TEM cells [8] and in vivo studies with animal models of AD [9], generating an interesting response that include attenuation of both cell function and disease manifestations. The first clinical trials with KV1.3 channels inhibitors are beginning to be planned in humans with promising results [10].

In this work we review the highlight of biological function of KV1.3 channel in T cells, consequence of the pharmacological inhibition as well as the possible human therapeutical application in ADs.

## 2 Voltage-gated ion channel structure and evolution: a very efficient biological strategy

Voltage-gated ion channels are essential for the functioning of the nervous, musculoskeletal, cardiovascular, digestive, hematopoietic, immune systems, among others, and are of utmost importance in the evolution of eukaryotes [11]. These channels are found in some prokaryotes, and their roles in these organisms are poorly known [12]. The superfamily of voltage-gated ion channels includes voltage-gated potassium channels (KVs), voltage-gated Ca2+ channels (CaVs) and voltage-gated sodium channels (NaVs), and are found in many living beings, suggesting that are extremely ancient [13].

KVs are tetramers composed of units, and each unit has 6 transmembrane subunits. T1 located at the cytoplasmic *N*-terminus [14,15] with a pore loop (*P*-loop) and a voltage-gated proton channel [16].

The functional heterogeneity of KVs arises from the multiple genes, splice variants and combinatorial of polypeptides to form heteromultimeric channels [17]. The amino acid residues TVGYG located at the *P*-loop of all four units is highly conserved [18]. Metazoan KVs channel are inactivated through occlusion of *P*-loop [19] or conformational change [20]. Interestingly, these channels fulfill essential functions in mammal neurons [21], cardio myocytes [22] and cells of the immune system [23], among others, whose main function is to balance the Vm with the potassium output of the cell in response to increase of intracytoplasmic Ca2+.

Some KVs channels evolved into CaVs channels, which are involved with the development of movement as such as in ciliates [24]. Early in eukaryotic evolution, the cells acquired a mechanism through channels to maintain an adequate intracellular concentration of Ca2+, not only to avoid its toxicity, but also to participate in effects associated with signaling capacity, mechanism that probably have been developed early in evolution simultaneously with the formation of intracellular compartments and vesicle trafficking [25].

Possibly Nav channels were derived from CaV channels [26]. NaVs are responsible of electrical signaling in excitable cells and are found in most bilaterians and believed to have evolved at the origin of the nervous system (i.e., sponges and placozoans) [27,28]. NaVs have a beta-subunit, present in mammalian vertebrates; the gene for this beta-subunit is found in the zebrafish (*Danio rerio*), but the protein is not expressed, which constitutes a link in the evolutionary process [29].

## 3 Ion channels in cells of the immune system

Cells of the immune systems express various ion channels necessary for different cellular functions, achieving different ion concentrations in the intracellular compartments. The most important regulations are related to the influx of Na+, Ca2+, Mg2+ and Zn2+ or efflux of K+ and Cl− [30]. In the context of the immune system, one of the most important functions are related to the activation of proteins involved in intracellular signaling.

Antigen presenting cells (APCs) such as dendritic cells or B cells, utilize three pathways of antigen (Ag) presentation. The Ag is presented to different T cell receptors (TCR)s. Major histocompatibility complex (MHC) class I molecules, presents Ag to the CD8^+^ T cell, MHC class II molecules to CD4^+^ T cell and CD1 molecules which are MHC class I-like molecules, present lipid Ags to diverse T cells [31]. The presentation of specific Ags to T cells by cell-to-cell interactions, results in the production of cytokines and additional APCs stimulation. The best understood cell signaling pathway family in APCs is that mediated by the mitogen-activated protein kinases (MAPK) [31]. The MAPK family consists of three main members: p38, ERK and JNK [32,33].

**Fig. 1:**
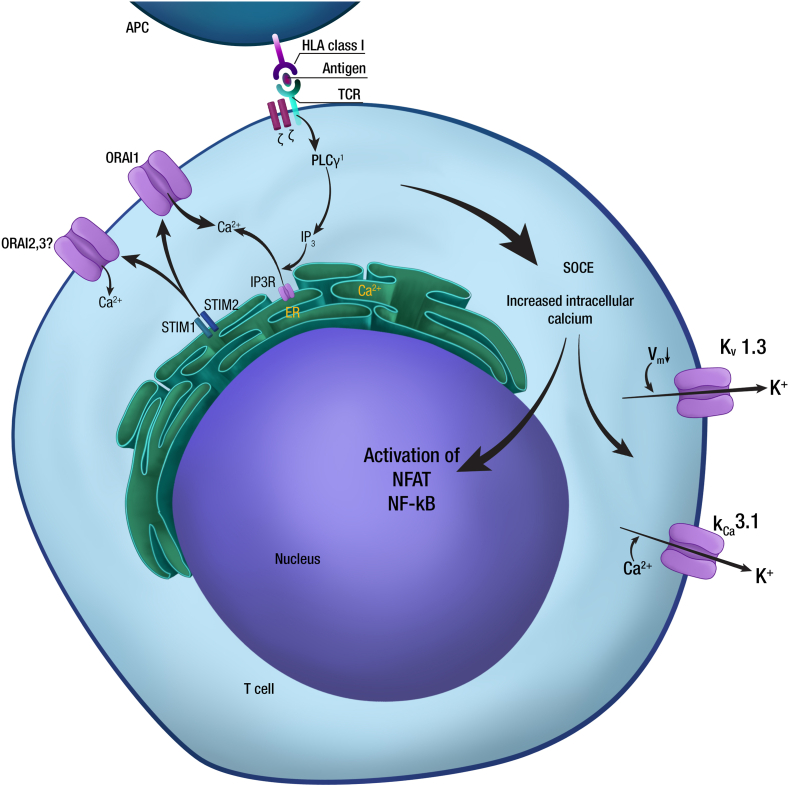
After antigen presentation from antigen presenting cells (APCs) trough major histocompatibility complex (MHC) class I molecules, the T cell receptor (TCR) activates tyrosine kinases which mediates increases in intracellular Ca2+ by activation of phospholipase C–γ1 (PLC-γ1), generating the second messenger's inositol 1,4,5-trisphosphate (IP3) and diacylglycerol (DAG) by hydrolysis of phosphatidylinositol-4,5-bisphosphate (PIP2). IP3, which binds to IP3 receptors (IP3R) in the endoplasmic reticulum (ER) membrane and triggers increases in intracellular Ca2+ from ER and activation of both nuclear factor of activated T cells (NFAT) and nuclear factor κB (NF-κB). Passage of Ca2+ from ER to the cytoplasm determines a reduction in the concentration of this ion in the ER, phenomena that activates stromal interaction molecules (STIM)1 and STIM2, which then translocate to ER-plasmatic membrane junctions, where they bind to ORAI1, a Ca2+ release–activated Ca2+ channel (CRAC) channel and mediates store-operated Ca2+ entry (SOCE). It generates even more the increase of intracytoplasmic Ca2+. Ca2+ influx depends additionally on membrane potential (Vm) which is restored by KV1.3 and KCa3. (Adapted from Feske et al., 2015).

After antigen presentation from the MHC molecules, the TCR activates tyrosine kinases, increasing intracellular Ca2+ and activating phospholipase C–γ1 (PLC-γ1) [34]. Activated PLC-γ1 generates the second messenger's inositol 1,4,5-trisphosphate (IP3) and diacylglycerol (DAG). IP3 joins IP3 receptors (IP3R) in the endoplasmic reticulum (ER) membrane and triggers Ca2+ flow from ER to cytoplasm [35], and its activates both nuclear factor κB (NF-κB) and nuclear factor of activated T cells (NFAT). DAG activates Ras-dependent signals, which are important for the induction of cytokines [36]. See Fig. 1.

The passage of Ca2+ from the ER to the cytoplasm, determines a reduction of this ion in the ER, phenomenon that derives in activates stromal interaction molecules (STIM)1 and STIM2, which then translocate ER to inner of plasmatic membrane. STIM1 and STIM2 join to ORAI1, a Ca2+ release–activated Ca2+ channel (CRAC) channel and mediates store-operated Ca2+ entry (SOCE), generating even more increase of intracytoplasmic Ca2+ [37]. SOCE is an essential mechanism regulating Ca2+ homeostasis [38]. T cells of CRAC channel deficient patients have a reduction of IL-2, IL-4, IL-10, IFNγ, TNFα, IL-17A and GM-CSF expression [37,39].

Ca2+ influx depends additionally on Vm established by two K+ channels, KV1.3 and KCa3.1. KV1.3 activation is generated by membrane depolarization, whereas KCa3.1 activation is generated by Ca2+ binding to the calmodulin [40]. See Fig. 1.

Other Ca2+ channels reported in T cells are P2X4, P2X7, transient receptor potential (TRPM)2 and TRPM7 among others. P2X4 is involved in T cell migration [41] and P2X7 inhibits regulatory T cell (Treg) differentiation [42]. Deletion of TRPM7 in murine T cells impairs cell development and function [43].

## 4 Immunologycal impact of ORAI1 and Kv1.3 channels inhibition

Dysregulated T cell response is characteristic of autoimmunity. The inhibition of T cells is a target of scientific research for potential medication development. Calcium signaling is fundamental for T cell effector function and calcium signaling inhibitors as cyclosporine A, partially satisfies the objective of adequately immunosuppressing of patients who require it and is not exempt of adverse reactions [44].

Pharmacological inhibition of ORAI1 with small molecules inhibitors have been tested in animal models [[45], [46], [47], [48], [49], [50], [51], [52]]. Few trials have been done in humans [53]. The inhibition of other ion channels being a major concern [54]. These inhibitors do not appear to be superior to medications such as ciclosporin A. Functional antibodies against human CRAC, have been developed and characterized for the neutralization of Ca2+ entry via CRAC channels [55,56]. Anti-Orai1 antibodies in vitro has been show inhibition of T cell proliferation and cytokine production in mouse model of graft-versus-host disease (GVHD) [57]. Human studies are lacking.

Autoreactive memory T cells are implicated in the pathogenesis of Ads, mainly CD4+CCR7−CD45RA− effector memory TEM cells phenotype, which have elevated Kv1.3 channel expression. This phenotype is observed in multiple sclerosis (MS) [58], type-1 diabetes mellitus (T1DM) and rheumatoid arthritis (RA) [59,60].

The development of immunosuppressants through blocking of Kv1.3 channel are based on toxins from the Caribbean Sea anemone (ShK toxin) (*Stichodactyla helianthus*) [61] and various scorpions as *Vaejovis mexicanus* (Vm24 toxin) [62], *Isometrus maculates* (ImKTx88 toxin, Imk) [63]*, Heterometrus spinifer* (HsTX1) [64], *Centruroides margaritatus* (margatoxin) [65] among others; monoclonal antibodies [66]; and nanoparticles with small interfering RNA (siRNA) [67].

Many toxins affect other related potassium channels (Kv1.1, Kv1.2, Kv1.6, Kv1.7) of neurons and muscle cells, potentially cause adverse effects [68]. Vm24 toxin from *V. mexicanus* is a potent and selective Kv1.3 channel blocker, an important finding for development of immunosuppressants for human [69,70]. Blockade of Kv1.3 channels with Vm24 does not affect the viability of TEM cells and inhibit the secretion of IFN-γ, TNF, IL-4, IL-5, IL-9, IL10, and IL-13 [62].

Kv1.3 inhibition with ShK suppress cytokine production, inhibits proliferation of TEM cells and ameliorates disease manifestation in animal models of delayed type hypersensitivity, T1DM, RA and MS [71]. HsTX1 is effective in control of pristane-induced arthritis model of RA [64]. Imk administration ameliorated experimental autoimmune encephalomyelitis severity [63].

From elsewhere, nanoparticles that selectively down-regulate Kv1.3 reduced CD40L and interferon-γ (IFNγ) in TEM cells from lupus nephritis patients in vitro [67].

On the other hand, taking into account both that naive and central memory T cells (TCM) up-regulate both Kv1.3 and KCa3.1 channels and that in autoimmune condition actived TEM cells by auto-antigen specific may selectively up-regulate Kv1.3 channels, with no significant up-regulation of KCa3.1 channels [72], a selective inhibition of Kv1.3 channels, without blockage of KCa3.1 channels, can selectively inhibit proliferation of TEM cells, without affecting naive and TCM cells. The use of blockers which can selectively inhibit Kv1.3 channels without inhibiting KCa3.1 channels or other important Kv channels (such as Kv1.1 or Kv1.5) can be a promising approach in treatment of T-cell mediated autoimmune diseases.

## 5 Clinical application of channel blockers

Dalazatide (ShK-186, SL5) is the first medication inhibitor of Kv1.3 channel used in human for the treatment of autoimmune condition as is the psoriasis. It is a synthetic peptide derivative of ShK [73]. In vivo studies with dalazatide is showed the inhibition of immune response of TEM cells without effect in naïve or central memory T cells [74]. Animals chronically treated with dalazatide do not show altered immunity against infections compared to dexamethasone [74].

A randomized phase 1b trial was conducted to evaluate both the safety and clinical response of repeat doses of dalazatide in adult patients with mild-to-moderate plaque psoriasis [10], showing that this medication was well tolerated and improve psoriatic skin lesions. Additionally, dalazatide reduced inflammation markers.

From elsewhere, over-activated T cells produce pro-inflammatory cytokines in pulmonary parenchyma, contributing substantially to the pathogenesis of the chronic obstructive pulmonary disease (COPD), concluding that inhibition of Kv1.3 channel can be an important therapeutic target [75]. Also, in inflammatory bowel disease (IBD) its usefulness has been postulated [76].

Immunohistochemical analysis of postmortem human brain of patient with Alzheimer's disease presents a significantly higher Kv1.3 staining intensity, leading to conclude that potential therapeutic targets could be the KV1.3 channel inhibition [77]. Based in animal models of MS, the therapeutic based in KV1.3 inhibition is promising [78]. Other autoimmune diseases such as RA or T1DM are also possible future therapeutic targets for Kv1.3 inhibitors, based on the knowledge of the pathogenesis of these diseases and the results of in vivo studies in animal models already mentioned.

## 6 Conclusions and perspectives

Inhibitors of Kv1.3 channels are an important tool both for the study of the pathogenesis of ADs and for the possible development of drugs for their management. Dalazatide, an inhibitor of these channels, showed safety and effectiveness in the treatment of patients with plaque psoriasis. New experimental models are necessary in this regard to answer different questions and give way to clinical studies in humans. Diseases such as RA, T1DM or MS will be targeted by these types of drugs, in the hope of achieving the best possible balance of effectiveness/safety.

## Author contributions

CAC, S C-V and F C-H wrote the manuscript and contributed to the medical observations. All authors read and approved the final manuscript.

## Funding

No sponsor is declared.

## Declaration of competing interest

The authors declare that they have no known competing financial interests or personal relationships that could have appeared to influence the work reported in this paper.
